# Acute prostatitis after prostate biopsy under ciprofloxacin prophylaxis with or without ornidazole and pre-biopsy enema: analysis of 3.479 prostate biopsy cases

**DOI:** 10.1590/S1677-5538.IBJU.2019.0257

**Published:** 2020-01-13

**Authors:** Muhsin Balaban, Orkunt Ozkaptan, Cuneyd Sevinc, Mustafa Yucel Boz, Rahim Horuz, Alper Kafkasli, Onder Canguven

**Affiliations:** 1 Department of Urology, Biruni University School of Medicine, Istanbul, Turkey; 2 Department of Urology, Kartal Training and Research Hospital, Istanbul, Turkey; 3 Department of Urology, Istinye University School of Medicine, Istanbul, Turkey; 4 Department of Urology, Medipol University School of Medicine, Istanbul, Turkey; 5 Department of Urology, Hamad Medical Corporation, Doha, Qatar; Weill Cornell Medicine, NY, USA

**Keywords:** Prostatitis, Prostate, Biopsy

## Abstract

**Objectives:**

To investigate the characteristics of cases of NIH category I acute prostatitis developed after transrectal prostate biopsy and clarifiy the risk factors and preventive factors.

**Materials and Methods:**

We retrospectively reviewed the medical records of 3.479 cases of transrectal ultrasound-guided needle biopsies performed with different prophylactic antibiotherapy regimens at two different institutions between January 2011 and February 2016. The patients of Group I have received ciprofl oxacin (n=1.523, 500mg twice daily) and the patients of Group II have received ciprofl oxacin plus ornidazole (n=1.956, 500mg twice daily) and cleansing enema combination as prophylactic antibiotherapy. The incidence, clinical features and other related microbiological and clinical data, were evaluated.

**Results:**

Mean age was 62.38±7.30 (47-75), and the mean prostate volume was 43.17±15.20 (21-100) mL. Of the 3.479 patients, 39 (1.1%) developed acute prostatitis after the prostate biopsy procedure. Of the 39 cases of acute prostatitis, 28/3.042 occurred after the first biopsy and 11/437 occurred after repeat biopsy (p=0.038). In Group I, 22 of 1.523 (1.4%) patients developed acute prostatitis. In Group II, 17 of 1.959 (0.8%) patients developed acute prostatitis. There was no statistical difference between the two groups according to acute prostatitis rates (X2=2.56, P=0.11). Further, hypertension or DM were not related to the development of acute prostatitis (P=0.76, X2=0.096 and P=0.83, X2=0.046, respectively).

**Conclusions:**

Repeat biopsy seems to increase the risk of acute prostatitis, while the use of antibiotics effective for anaerobic pathogens seems not to be essential yet.

## INTRODUCTION

Transrectal ultrasound-guided needle biopsy (TRUS-Bx) is generally accepted as a standard procedure for the diagnosis of prostate cancer (1). Despite various studies in the literature demonstrating low rates of complications and good tolerance to the procedure, it is still considered invasive and not entirely free of complications (2, 3). Pain, mild hematuria, or hemospermia are common, self-limited, minor complications (4). Urinary tract infection (UTI or bacteremia, however, are potentially severe complications (5). Prophylactic antibiotic therapy and prebiopsy enema are recommended for preventing infectious complications (6–8). Oral quinolones, either alone or in combination with other antibiotherapeutic agents, are the most common prophylactic practices. Unfortunately, in the last few years, increased resistance to quinolones has been reported in association with a rise in severe infectious complications after biopsy (9). In this study, we aimed to compare the incidence of prostatitis after prostate biopsy using two different prophylactic antibiotic protocols and provide an overview of the bacteriologic characteristics of urine and blood cultures, as well as antimicrobial resistance in NIH category I acute bacterial prostatitis.

## MATERIALS AND METHODS

We reviewed the medical records of 3.479 patients TRUS-Bx cases performed between January 2011 and February 2016 in two different urology clinics of the same tertiary referral centre. Of these 3.479 patients, 437 cases underwent a repeat biopsy in case of a negative initial biopsy. The patients were elected from a PSA screening program that was applied in our hospital for patients in the age of fifty and afterwards once a year. Patients who were classified as underweighted (20kg/m2> body mass index (BMI)) and morbid obese (>35kg/m2) were excluded. Further patients with uncontrolled diabetes mellitus were also excluded. A specific matching procedure was not applied.

None of the patients had signs and symptoms of UTI or acute prostatitis before TRUS-Bx. The indications for TRUS-Bx were a PSA level >4.0ng/mL and abnormal findings in TRUS or by digital rectal exam (DRE). All procedures were performed with an 18-gauge core biopsy needle mounted on an automatic biopsy gun (Geotek Medical, Turkey) under local anaesthesia and ten core specimens were obtained in all biopsies. A blood test for prothrombin time and activated partial thromboplastin time were performed for all patients to obtain a coagulation profile. Patients were instructed not to take any oral anticoagulants in the last seven days before the TRUS-Bx procedure. Prophylactic treatment for TRUS-Bx was applied in two different regimens. The biopsy protocols were identical throughout the study period except for the prophylaxis regimen. Group I (n=1.523) received ciprofloxacin alone (500mg twice daily) continually for three days. Group II (n=1.956) received ciprofloxacin (500mg twice daily) plus ornidazole (500mg twice daily) for three days in addition to cleansing enema. Cleansing enema was applied one hour before the procedure.

All biopsies were performed in an outpatient department biopsy room by two doctors of each group. Acute prostatitis was diagnosed by a body temperature >38°C, positive urine analysis (bacteriuria and/or pyuria >10^3^/mL), and pathologic, clinical findings by DRE. 5 Patients who developed acute prostatitis were admitted to hospital for treatment. Blood and urine samples were collected for bacterial evaluation before antimicrobial treatment was initiated. All organisms isolated from blood and urine cultures were tested for antimicrobial susceptibility. Informed consent was obtained from all patients before the biopsy procedure, and institutional approval was obtained for the study.

Cross-analysis with the chi-square test was performed to confirm the incidence rates of complications and their correlation between prophylactic treatment with only ciprofloxacin or ciprofloxacin plus ornidazole and cleansing enema. All statistical tests were two-sided with the significance level set at P <0.05.

## RESULTS

Mean age of the patients was 62.38±7.30 (47-75) years. Descriptive statistics for the two different groups are presented in Table-1. Of the 3.479 patients, 39 (1.1%) patients developed acute prostatitis with symptoms within 1.28 (0-4) days after prostate biopsy. The patients with acute prostatitis symptoms were hospitalized and treated with two different intravenous antibiotics, including a third-generation cephalosporin (ceftriaxone) and an aminoglycoside (gentamicin) after urine and blood samples were taken for culture. The mean hospitalization time of acute prostatitis patients was 4.76±3.20 (2–20) days. Of the 39 patients, 15 (38%) had positive urine cultures, and of those, 13 (33%) had positive blood cultures. Both urine and blood cultures were negative in 24 (61%) of infected patients. Extended-spectrum beta-lactamase (ESBL)-producing E. coli were detected in 10 patients. Septic shock was observed in no patient and all patients cured after treatment.

**Table 1:** Descriptive Statistics.

Variables	Ciprofloxacin group	Ciprofloxacin+ornidazole+ cleansing enema group	P-value
Age, year; mean ±SD (range)	62.1±7.4 (47-75)	62.34±7.19 (49-75)	0.61
BMI, kg/m^2^; mean ±SD (range)	29.12±2.9 (20.7-33.8)	28.98±3.2 (20.3-34.4)	0.76
Prostate volume, mL; mean ±SD (range)	43.1±14.8 (21-91)	44.5±15.9 (22-100)	0.45
Preoperative PSA, ng/dL; mean±SD (range)	7.12±3.4 (2.4-95)	7.8±3.7(1.8-59)	0.53

The most frequent comorbidities were hypertension and DM (25.1% (n=875) and 16.7% (n=580), respectively). The rate of hypertension and DM in patients who developed acute prostatitis was 23% (n=9) and 17.9% (n=7), respectively. Hypertension or DM were not related to the development of acute prostatitis (P=0.76, X2=0.096 and P=0.83, X2=0.046, respectively). In Group I, in which only ciprofloxacin was used for prophylaxis, 22 of 1.522 (1.4%) patients developed acute prostatitis. In Group II, in which ciprofloxacin plus ornidazole and cleansing enema were used, 17 of 1.957 (0.8%) patients developed acute prostatitis. There was no statistical difference between the two groups according to acute prostatitis rate (X2=2.56, P=0.11). Of the 3.479 patients, 437 underwent a second biopsy. Acute prostatitis occurred after first and repeated biopsies in 28 (0.92%) and 11 (2.5%) patients, respectively (Table-2). The difference between the latter first and repeat biopsy groups according to the rate of acute prostatitis was statistically significant (P=0.001, X2=11.55).

**Table 2:** Analysis of acute prostatitis cases in different prophylactic treatment regimes of transrectal postate biopsy. ESBL-Producing *E. Coli:* Extended-spectrum Beta-lactamase and Quinolone-resistant *Escherichia Coli*.

	Ciprofloxacin group	Ciprofloxacin+ornidazole+ cleansing enema group
Acute Prostatitis, n (%)	22 (1.4)	17 (0.8)
Symptoms starting time, days	1.28	1.32
Hospitalization time, day (mean±SD)	4.76±3.20	4.87±3.40
First Prostate Biopsy (n/N)	16/1320	12/1722
Second Prostate Biopsy (n/N)	6/203	5/234
Comorbidities		
HT	5	4
DM	4	3
Bacteria Culture		
Positive urinary culture, n (%)	7 (31.8)	8 (47)
Positive blood culture, n (%)	6(27.2)	7 (41)
ESBL- Producing *E. Coli*, n (%)	4 (18)	6 (35)
Blood culture ±	3/1	5/1

## DISCUSSION

In this study, we investigated the incidence of acute prostatitis under two different prophylactic regimens (ciprofloxacin alone or ciprofloxacin plus ornidazole with a cleansing enema) with bacteriologic characteristics of blood and urine cultures. Of the 3.479 patients, 39 (1.1%) developed acute prostatitis. Of these 39 patients, 15 (38%) had positive urine cultures, and 8 (20%) had positive blood cultures. According to our study, the rate of acute prostatitis was not statistically different between the two groups. Fluoroquinolones are one of the most effective antibiotics for the genitourinary system and show excellent penetration into the prostate tissue, and because the vast majority of uropathogens and enteric species have proper susceptibility to these agents, most of the trials have focused on fluoroquinolones (10–12). In the current study, ciprofloxacin was used as a prophylactic drug for Group I patients. Although frequently encountered uropathogens and coliforms such as E.coli or Klebsiella spp are usually responsible for the incidence of infectious complications after prostate biopsy, some infrequently encountered enteric pathogens, especially anaerobes, were reported in fever and septicemia after prostate biopsy, and the effects in the majority of these cases were severe and devasting (13, 14). Therefore, many studies have been conducted in the field of anaerobic coverage by prophylactic antibiotics during prostate biopsy (15, 16). Hence, as anaerobic bacteria coverage drug ornidazole was added to the ciprofloxacin prophylaxis therapy for Group II. It is widely accepted that antibiotic prophylaxis before TRUS-Bx is effective in preventing infectious complications (4). Despite the use of prophylactic antibiotics, bacterial infections causing fever, urinary tract infection, acute prostatitis and orchioepididymitis occur in 1-5% of patients (17, 18) Therefore, many researchers have attempted to identify the optimal prophylactic regimen to use peri-procedure, i.e. TRUS-Bx to decrease the risks of infectious complications. In this study, we investigated the incidence of acute prostatitis under two different prophylactic regimens (ciprofloxacin alone or ciprofloxacin plus ornidazole with a cleansing enema) with bacteriologic characteristics of blood and urine cultures. Intrarectal flora is disseminated within the prostate due to the insertion of biopsy equipment into the prostate through the rectum during TRUS-Bx. Theoretically, using prophylactic treatment for colonic flora may prevent infectious complications of the prostate. Fluoroquinolones (ciprofloxacin) are used most commonly as antibiotic prophylaxis due to their broad spectrum of antibacterial activity (17, 19). Other features include coverage for most aerobic microorganisms residing in the bowel, good oral bioavailability (70%-80%), extended half-life (4-5 hours), high concentration in both urine and prostate tissue, and post-antibiotic suppression of bacterial growth lasting for two to six hours (17). These features make ciprofloxacin a logical candidate to prevent prostatitis after prostate biopsy, however, the spread of fluoroquinolone resistance threatens to undermine the risk-benefit profile in transrectal prostate biopsy. The American Urological Association recommends prophylactic antibiotics within 24 hours of prostate biopsy for all patients, with a fluoroquinolone the antibiotic of choice (19). However, several studies have reported that acute prostatitis after TRUS-biopsy continues to occur, ranging from 1.3 to 9.3% (20, 21). In our study, ciprofloxacin was used as prophylactic treatment within 24 hours of prostate biopsy procedure and continued for three days after biopsy in Group Ḭ. Of these 1.523 patients, (22) (1.4%) developed acute prostatitis and were admitted to hospital for treatment within 1.28 (0–4) days following the prostate biopsy. Prophylactic antibiotic therapy and prebiopsy enema are recommended for preventing infectious complications (6). Ghafoori et al. conducted a study on 208 patients by categorizing them into either a povidone-iodine enema group or a control (no enema) group. A significant decrease in infectious complications was observed in patients who received an enema (22). Kam et al. designed a study using 1.083 patients in which 658 (60.8%) had a prebiopsy enema in addition to prophylactic antibiotic treatment. Prebiopsy enema was done using glycerin or saline. Among these, 31 (4.7%) experienced complications. A prebiopsy enema was not performed in 425 patients (39.8%), of whom 38 (8.9%) had complications (p=0.007) (23). According to our study we only used fluoroquinolone in Group 1, nevertheless we found no significant difference in prostatitis rates between the two groups.

Ornidazole is a 5-nitroimidazole derivative and effective for anaerobic enteric pathogens, and it can be used as a prophylactic treatment before rectal surgery (24). In our study, ciprofloxacin, ornidazole, and cleansing enema were used as a prophylactic treatment in Group II patients, where 17 of 1.959 patients (0.8%) developed acute prostatitis. In this group, the prebiopsy enema was done using glycerin or saline. A Cochrane review concluded that enema plus antibiotics reduced the risk of bacteremia (relative risk [RR]: 0.25, 95% CI, 0.08-0.75) compared with antibiotics alone, but no differences were found in the rate of fever, or infection (25). The current study revealed no statistical difference according to acute prostatitis rates. In the presented study, 3.042 patients underwent a biopsy for the first time, and 437 had repeat biopsies. Acute prostatitis developed in 28 (0.92%) patients in the primary biopsy group and 11 (2.5%) patients in the repeat biopsy group. The risk of acute prostatitis was significantly higher after repeated biopsies. Similarly, Shigehara et al. investigated 457 patients, 371 underwent primary biopsy and 86 underwent repeat biopsy. Overall, acute bacterial prostatitis developed in six patients (1.3%). The rate of acute prostatitis after initial and repeat biopsies was 0.5% (n=2) and 4.7% (n=4). This result was statistically significant (26). Further Ozden et al. reported that a greater rate of acute prostatitis was observed in their repeat biopsy group than in patients who had undergone a prostate biopsy procedure for the first time (6.8% vs. 1.3%) (20). These studies concluded that repeat biopsies can be a risk factor for acute prostatitis. New biopsy technologies such as magnetic resonance imaging-transrectal ultrasonography (MRI-TRUS) fusion-guided-3D targeted biopsies have the potential to reduce the number of repeated biopsies (27). In cases of acute prostatitis, it is important to take blood and urine samples for culture. Song et al. reported that 63 of the 103 acute prostatitis cases (61.1%) had positive blood and/ or urine cultures. In our study, the rate of positive urine and/or blood cultures was 58.9%, this result was higher compared with literature. We could not make a rational explanation for this difference.

Another critical concern is the emergence of quinolone-resistant Escherichia Coli (*E.coli*) and extended-spectrum beta-lactamases (ESBL)-producing E.coli strains. Song et al. reviewed 11.345 TRUS-Bx cases, and 63 cases of acute prostatitis were identified. ESBL producing E. coli strains were detected in 12 patients (20%). Three of the 12 patients had to be admitted to the intensive care unit and were treated successfully with intravenous ertapenem (28) In our study cohort, 39 patients developed infective symptoms after prostate biopsy.

Suzuki et al. conducted a study on DM patients in which they performed prostate biopsy using the transperineal approach. They concluded that transperineal biopsy could be carried out without major infectious complications in DM men (29). In our study population, seven of 39 prostatitis patients had DM. Our results revealed that DM was not related to acute prostatitis. Besides, hypertension was also not associated with the occurrence of acute prostatitis.

A limitation of the study is that the it involves two different cohorts from two different clinics, as a result, slight diagnostic difference may have affected the results. Another weakness is that some patients in which complications occurred could have been be treated in other hospitals. Further, the retrospective design is a limitation. More reliable results would be obtained in prospective randomized study design.

In conclusion, repeat biopsies can increase the acute prostatitis risk after TRUS-Bx. The use of prebiopsy cleansing enema with ornidazole treatment in addition to ciprofloxacin prophylaxis did not decrease the acute prostatitis risk. Although resistance to fluoroquinolones has increased over the years, the use of antibiotics effective for anaerobic pathogens seems not to be essential yet.

## References

[1] 1. Heidenreich A, Bellmunt J, Bolla M, Joniau S, Mason M, Matveev V, et al. EAU guidelines on prostate cancer. Part 1: screening, diagnosis, and treatment of clinically localised disease. Eur Urol. 2011;59:61-71. 10.1016/j.eururo.2010.10.039 21056534

[2] 2. Shigemura K, Tanaka K, Yasuda M, Ishihara S, Muratani T, Deguchi T, et al. Efficacy of 1-day prophylaxis medication with fluoroquinolone for prostate biopsy. World J Urol. 2005;23:356-60. 10.1007/s00345-005-0024-4 16254727

[3] 3. Tobias-Machado M, Corrêa TD, De Barros EL, Wroclawski ER. Antibiotic prophylaxis in prostate biopsy. A comparative randomized clinical assay between ciprofloxacin, norfloxacin and chloramphenicol. Int Braz J Urol. 2003;29:313-9. 10.1590/s1677-55382003000400005 15745554

[4] 4. Campeggi A, Ouzaid I, Xylinas E, Lesprit P, Hoznek A, Vordos D, et al. Acute bacterial prostatitis after transrectal ultrasound-guided prostate biopsy: epidemiological, bacteria and treatment patterns from a 4-year prospective study. Int J Urol. 2014;21:152-5. 10.1111/iju.12207 23906113

[5] 5. Djavan B, Waldert M, Zlotta A, Dobronski P, Seitz C, Remzi M, et al. Safety and morbidity of first and repeat transrectal ultrasound guided prostate needle biopsies: results of a prospective European prostate cancer detection study. J Urol. 2001;166:856-60. 11490233

[6] 6. Rodríguez LV, Terris MK. Risks and complications of transrectal ultrasound guided prostate needle biopsy: a prospective study and review of the literature. J Urol. 1998;160(6 Pt 1):2115-20. 10.1097/00005392-199812010-00045 9817335

[7] 7. Kim SJ, Kim SI, Ahn HS, Choi JB, Kim YS, Kim SJ. Risk factors for acute prostatitis after transrectal biopsy of the prostate. Korean J Urol. 2010;51:426-30. 10.4111/kju.2010.51.6.426 PMC2890061 20577611

[8] 8. Brown RW, Warner JJ, Turner BI, Harris LF, Alford RH. Bacteremia and bacteriuria after transrectal prostatic biopsy. Urology. 1981;18:145-8. 10.1016/0090-4295(81)90425-8 7269016

[9] 9. Al-Hasan MN, Lahr BD, Eckel-Passow JE, Baddour LM. Antimicrobial resistance trends of Escherichia coli bloodstream isolates: a population-based study, 1998-2007. J Antimicrob Chemother. 2009;64:169-74. 10.1093/jac/dkp162 PMC2692504 19435736

[10] 10. Lugg J, Lettieri J, Stass H, Agarwal V. Determination of the concentration of ciprofloxacin in prostate tissue following administration of a single, 1000 mg, extended-release dose. J Chemother. 2008;20:213-8. 10.1179/joc.2008.20.2.213 18467248

[11] 11. Lindstedt S, Lindström U, Ljunggren E, Wullt B, Grabe M. Single-dose antibiotic prophylaxis in core prostate biopsy: Impact of timing and identification of risk factors. Eur Urol. 2006;50:832-7. 10.1016/j.eururo.2006.05.003 16750292

[12] 12. Naber KG, Sörgel F. Antibiotic therapy––rationale and evidence for optimal drug concentrations in prostatic and seminal fluid and in prostatic tissue. Andrologia. 2003;35:331-5. 14535866

[13] 13. Webb NR, Woo HH. Antibiotic prophylaxis for prostate biopsy. BJU Int. 2002;89:824-8. 10.1046/j.1464-410x.2002.02735.x 11972504

[14] 14. Brewster SF, Rooney N, Kabala J, Feneley RC. Fatal anaerobic infection following transrectal biopsy of a rare prostatic tumour. Br J Urol. 1993;72:977-8. 10.1111/j.1464-410x.1993.tb16316.x 8306172

[15] 15. Aron M, Rajeev TP, Gupta NP. Antibiotic prophylaxis for transrectal needle biopsy of the prostate: a randomized controlled study. BJU Int. 2000;85:682-5. 10.1046/j.1464-410x.2000.00576.x 10759665

[16] 16. Breslin JA, Turner BI, Faber RB, Rhamy RK. Anaerobic infection as a consequence of transrectal prostatic biopsy. J Urol. 1978;120:502-3. 10.1016/s0022-5347(17)57246-8 702677

[17] 17. Kapoor DA, Klimberg IW, Malek GH, Wegenke JD, Cox CE, Patterson AL, et al. Single-dose oral ciprofloxacin versus placebo for prophylaxis during transrectal prostate biopsy Urology. 1998;52:552-8. 10.1016/s0090-4295(98)00296-9 9763070

[18] 18. Rietbergen JB, Kruger AE, Kranse R, Schröder FH Complications of transrectal ultrasound-guided systematic sextant biopsies of the prostate: evaluation of complication rates and risk factors within a population-based screening program. Urology. 1997;49:875-80. 10.1016/s0090-4295(97)00100-3 9187694

[19] 19. Wolf JS Jr, Bennett CJ, Dmochowski RR, Hollenbeck BK, Pearle MS, Schaeffer AJ, et al. Best practice policy statement on urologic surgery antimicrobial prophylaxis. J Urol 2008;179:1379-90. 10.1016/j.juro.2008.01.068 18280509

[20] 20. Ozden E, Bostanci Y, Yakupoglu KY, Akdeniz E, Yilmaz AF, Tulek N, et al. Incidence of acute prostatitis caused by extendedspectrum beta-lactamase-producing Escherichia coli after transrectal prostate biopsy. Urology. 2009;74:119-23. 10.1016/j.urology.2008.12.067 19464043

[21] 21. Mosharafa AA, Torky MH, El Said WM, Meshref A. Rising incidence of acute prostatitis following prostate biopsy: fluoroquinolone resistance and exposure is a significant risk factor. Urology. 2011;78:511-4. 10.1016/j.urology.2011.04.064 21782225

[22] 22. Ghafoori M, Shakiba M, Seifmanesh H, Hoseini K. Decrease in infection rate following use of povidone-iodine during transrectal ultrasound guided biopsy of the prostate: a double blind randomized clinical trial. Iran J Radiol. 2012;9:67-70. 10.5812/iranjradiol.7561 PMC3522353 23329966

[23] 23. Kam SC, Choi SM, Yoon S, Choi JH, Lee SH, Hwa JS, et al. Complications of transrectal ultrasound-guided prostate biopsy: impact of prebiopsy enema. Korean J Urol 2014;55:732-6. 10.4111/kju.2014.55.11.732 PMC4231150 25405015

[24] 24. McArdle CS, Morran CG, Pettit L, Gemmell CG, Sleigh JD, Tillotson GS. Value of oral antibiotic prophylaxis in colorectal surgery. Br J Surg. 1995;82:1046-8. 10.1002/bjs.1800820814 7648148

[25] 25. Zani EL, Clark OA, Rodrigues Netto N Jr. Antibiotic prophylaxis for transrectal prostate biopsy. Cochrane Database Syst Rev. 2011;(5):CD006576. 10.1002/14651858.CD006576.pub2 PMC12301957 21563156

[26] 26. Shigehara K, Miyagi T, Nakashima T, Shimamura M. Acute bacterial prostatitis after transrectal prostate needle biopsy: clinical analysis. J Infect Chemother. 2008;14:40-3. 10.1007/s10156-007-0570-3 18297448

[27] 27. Sonn GA, Natarajan S, Margolis DJ, MacAiran M, Lieu P, Huang J, et al. Targeted biopsy in the detection of prostate cancer using an office based magnetic resonance ultrasound fusion device. J Urol. 2013;189:86-91. 10.1016/j.juro.2012.08.095 PMC3561472 23158413

[28] 28. Song W, Choo SH, Sung HH, Han DH, Jeong BC, Seo SI, et al. Incidence and management of extended-spectrum beta-lactamase and quinolone-resistant Escherichia coli infections after prostate biopsy. Urology. 2014;84:1001-7. 10.1016/j.urology.2014.06.052 25443894

[29] 29. Suzuki M, Kawakami S, Asano T, Masuda H, Saito K, Koga F, et al. Safety of transperineal 14-core systematic prostate biopsy in diabetic men. Int J Urol. 2009;16:930-5. 10.1111/j.1442-2042.2009.02386.x 19796129

